# Acanthocytosis and HyperCKemia

**DOI:** 10.4274/tjh.2017.0142

**Published:** 2018-11-13

**Authors:** Uluç Yiş, Kerstin Becker, Şebnem Yılmaz, Sebahattin Çırak

**Affiliations:** 1 Dokuz Eylül University Faculty of Medicine, Department of Pediatrics, Division of Pediatric Neurology, İzmir, Turkey; 2 University Hospital Cologne, Department of Pediatrics, Cologne, Germany; 3 University of Cologne, Center for Molecular Medicine Cologne, Cologne, Germany; 4 Dokuz Eylül University Faculty of Medicine, Department of Pediatrics, Division of Pediatric Hematology, İzmir, Turkey

**Keywords:** Acathocytosis, Elevated creatine kinase, Muscle disease

A 14-year-old boy was referred to the neuromuscular clinic for the investigation of hyperCKemia (serum creatinine kinase: 4000 IU/L; normal range: 0-170), detected during laboratory examinations. He was the first child of consanguineous parents. His psychomotor development was normal and he had no past symptoms of a neuromuscular disease. Detailed history taking did not reveal any signs of involuntary movements, bradykinesia, or social problems. Neurologic examination showed normal muscle power in the upper and lower extremities and no signs of muscle atrophy. Deep tendon reflexes could not be elicited. Nerve conduction studies were normal but electromyography revealed combined neurogenic and myogenic potentials in the lower extremity muscles. A muscle biopsy did not show any pathology and was interpreted as normal. Targeted customized Mendeliome panel [1] next-generation sequencing revealed a homozygous splice site mutation in the vacuolar protein sorting-associated protein *(VPS13A)*, NM_015186.3, c.6095+1G>C.

HyperCKemia is a condition characterized by elevated levels of the enzyme creatinine kinase in the blood. Chorea-acanthocytosis is an autosomal recessive disease caused by mutations in the *VPS13A* gene, which encodes the protein chorein. The disease is characterized by chorea, dystonias mainly involving the face, parkinsonism, vocal tics, epilepsy, social disinhibition, and distal muscle wasting. The mean age of onset is 35 years [2]. Neuropsychiatric symptoms are also common and may precede movement disorders. Acanthocytes usually constitute 5% to 50% of circulating red blood cells. They may also be absent or may appear late in the course of the disease [3]. Most patients have elevated serum creatinine kinase levels but the cause of this creatinine kinase elevation is unknown. Nerve conduction studies may be normal, but may show sensory axonal neuropathy in some cases. Electromyography may show myogenic or neurogenic potentials. Retrospectively, after the genetic diagnosis, we could confirm the presence of acanthocytes (Figure 1). Our patient had no neurological complaints and no neurological abnormalities. Thus, peripheral blood smears may give important diagnostic clues in cases of idiopathic hyperCKemia. Whole exome sequencing is also a preferable diagnostic modality in cases of idiopathic hyperCKemia, but there are challenges in the counseling of the family in a clinically asymptomatic case in the context of a progressive neurologic disorder.

## Figures and Tables

**Figure 1:**
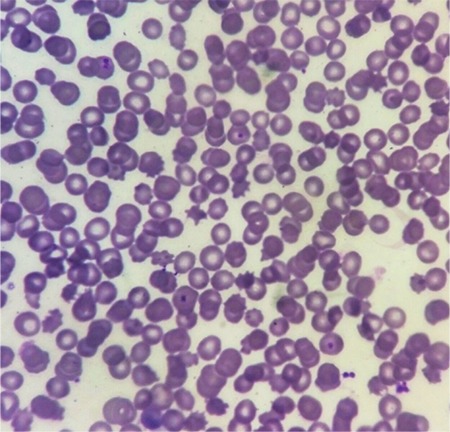
Peripheral blood smear of the patient showing acanthocytosis.
